# Serum-derived extracellular vesicles from breast cancer patients contribute to differential regulation of T-cell-mediated immune-escape mechanisms in breast cancer subtypes

**DOI:** 10.3389/fimmu.2023.1204224

**Published:** 2023-06-22

**Authors:** Rosalind Graham, Patrycja Gazinska, Birong Zhang, Atousa Khiabany, Shubhankar Sinha, Thanussuyah Alaguthurai, Fabian Flores-Borja, Jose Vicencio, Fabienne Beuron, Ioannis Roxanis, Rafal Matkowski, Revadee Liam-Or, Andrew Tutt, Tony Ng, Khuloud T. Al-Jamal, You Zhou, Sheeba Irshad

**Affiliations:** ^1^ Breast Immunology Group, School of Cancer & Pharmaceutical Sciences, King’s College London, London, United Kingdom; ^2^ Breast Cancer Now Research Unit, King's College London, Guy's Hospital, London, United Kingdom; ^3^ Breast Cancer Now Toby Robins Research Centre, Institute of Cancer Research, London, United Kingdom; ^4^ Biobank Research Group, Lukasiewicz Research Network – PORT Polish Center for Technology Development, Wroclaw, Poland; ^5^ Systems Immunity University Research Institute and Division of Infection and Immunity, School of Medicine, Cardiff University, Cardiff, United Kingdom; ^6^ Richard Dimbleby Laboratory of Cancer Research School of Cancer and Pharmaceutical Sciences, King’s College London, London, United Kingdom; ^7^ UCL Cancer Institute, Paul O'Gorman Building, University College London, London, United Kingdom; ^8^ Breast Unit, Lower Silesian Oncology, Pulmunology and Hematology Center, Wroclaw, Poland; ^9^ Department of Oncology, Wroclaw Medical University, Wroclaw, Poland; ^10^ Institute of Pharmaceutical Science, Faculty of Life Sciences & Medicine, King's College London, London, United Kingdom; ^11^ Medical Oncology, Guy's & St Thomas' NHS Trust, London, United Kingdom

**Keywords:** extracellular vesicles, breast cancer, T cells, immune regulation, tumour microenvironment

## Abstract

**Background:**

Intracellular communication within the tumour is complex and extracellular vesicles (EVs) have been identified as major contributing factors for the cell-to-cell communication in the local and distant tumour environments. Here, we examine the differential effects of breast cancer (BC) subtype-specific patient serum and cell-line derived EVs in the regulation of T cell mediated immune responses.

**Methods:**

Ultracentrifugation was used to isolate EVs from sera of 63 BC patients, 15 healthy volunteers and 4 human breast cancer cell lines. Longitudinal blood draws for EV isolation for patients on neoadjuvant chemotherapy was also performed. Characterization of EVs was performed by Nanoparticle Tracking Analysis (NTA), transmission electron microscopy (TEM) and immunoblotting. CD63 staining was performed on a tissue microarray of 218 BC patients. In-house bioinformatics algorithms were utilized for the computation of EV associated expression scores within The Cancer Genome Atlas (TCGA) and correlated with tumour infiltrating lymphocyte (TIL) scores. *In vitro* stimulation of PBMCs with EVs from serum and cell-line derived EVs was performed and changes in the immune phenotypes characterized by flow cytometry. Cytokine profiles were assessed using a 105-plex immunoassay or IL10 ELISA.

**Results:**

Patients with triple negative breast cancers (TNBCs) exhibited the lowest number of EVs in the sera; whilst the highest was detected in ER+HER2+ cancers; reflected also in the higher level of CD63+ vesicles found within the ER+HER2+ local tumour microenvironment. Transcriptomic analysis of the TCGA data identified that samples assigned with lower EV scores had significantly higher abundance of CD4+ memory activated T cells, T follicular cells and CD8 T cells, plasma, and memory B cells; whilst samples with high EV scores were more enriched for anti-inflammatory M2 macrophages and mast cells. A negative correlation between EV expression scores and stromal TIL counts was also observed. In vitro experiments confirmed that circulating EVs within breast cancer subtypes have functionally differing immunomodulatory capabilities, with EVs from patients with the most aggressive breast cancer subtype (TNBCs) demonstrating the most immune-suppressive phenotype (decreased CD3+HLA-DR+ but increased CD3+PD-L1 T cells, increased CD4+CD127-CD25hi T regulatory cells with associated increase in IL10 cytokine production). In depth assessment of the cytokine modulation triggered by the serum/cell line derived exosomes confirmed differential inflammatory cytokine profiles across differing breast cancer subtypes. Studies using the MDA-231 TNBC breast cancer cell-line derived EVs provided further support that TNBC EVs induced the most immunosuppressive response within PBMCs.

**Discussion:**

Our study supports further investigations into how tumour derived EVs are a mechanism that cancers can exploit to promote immune suppression; and breast cancer subtypes produce EVs with differing immunomodulatory capabilities. Understanding the intracellular/extracellular pathways implicated in alteration from active to suppressed immune state may provide a promising way forward for restoring immune competence in specific breast cancer patient populations.

## Introduction

Breast cancer (BC) remains the most prevalent type of cancer occurring in women worldwide, making it a leading cause of cancer‐related death globally (1, 2). BC cells exhibit formidable molecular heterogeneity and are classically subtyped based on hormone receptors (HR; oestrogen and progesterone receptors) and human epidermal growth factor receptors (HER2). In general, patients with HR^+^ and HER2^−^ cancers have better prognoses compared with those with HR^−^HER2^+^ or triple-negative cancers (ER^−^PR^−^HER2^−^; TNBC), which are more aggressive (3, 4). It is increasingly being well-recognised that molecular mechanisms employed by BC cells to subvert or escape from immune recognition play an important role in BC development, progression, and sensitivity to therapies (5). Additionally, the tumour microenvironment (TME) of breast tumours (tissue-specific resident and recruited stromal cell types) varies based on the tumour cell subtype, with TNBC being recognised as a more immunogenic breast cancer (6). The presence of tumour-infiltrating lymphocytes (TILs) is correlated with a good prognosis and outcome in triple-negative and HER2^+^ BC. In contrast, emerging evidence points to high immune infiltration in hormone-receptor-positive cancers being associated with unfavourable outcomes (7). Intracellular communication within the tumour is complex, and extracellular vesicles (EVs) have been identified as major contributing factors for cell-to-cell communication in the local and distant tumour environments (8).

EVs are heterogeneous submicron-sized vesicles that include microvesicles (MVs) (0.1–2 μm), apoptotic bodies (1–5 μm), and exosomes (30–150 nm). Recently, novel subpopulations of exosomes (large exosome vesicles, 90–120 nm; small exosome vesicles, 60–80 nm) and an abundant population of nonmembranous nanoparticles termed “exomeres” (~35 nm) have been identified (9, 10). Some size overlap does exist, however. Recent evidence suggests cancer-derived exosomes and oncosomes (atypically large 1–10 µm diameter) can have dichotomous roles in the regulation of the immune system, enhancing or suppressing an immune response depending on the cell of origin, target cell, and its functional state (11–13). There is growing evidence that exosomes also contribute to the remodelling of tumour immune microenvironments. Cancer cells can evade antitumour immunity by packaging PD-L1 into their exosomes, and exosomal PD-L1 inhibits T-cell activation, allowing them to evade antitumour immunity (14). In addition, exosomal PD-L1 appears to be resistant to anti-PD-L1 antibody blockade (15). However, PD-L1 expression is heterogeneous and dynamic among different BCs. In the context of breast cancer heterogeneity, profiling of breast cancer cell-line-derived EVs has demonstrated that vesicular content is diverse in nature and varies depending on the parent cell. For example, TNBC cell-line MDA-MB-231 EVs are enriched in the beta chain of MHC Class I molecules, supporting the involvement of breast cancer EVs in altering immune system recognition to promote cancer growth (16).

Here, we report that patients with triple-negative breast cancers (TNBCs) exhibited the lowest number of extracellular vesicles in the sera, whilst the highest was detected in ER^+^HER2^+^ cancers, reflected also in the higher level of CD63^+^ vesicles found within the ER^+^HER2^+^ local tumour microenvironment. *In vitro* experiments confirmed that c**i**rculating EVs within breast cancer subtypes have functionally differing immunomodulatory capabilities, with EVs from patients with TNBC cancers demonstrating the most immune-suppressive phenotype.

## Results

### Higher serum-derived extracellular vesicles isolated from HER2^+^ breast cancers

**Figure 1:**
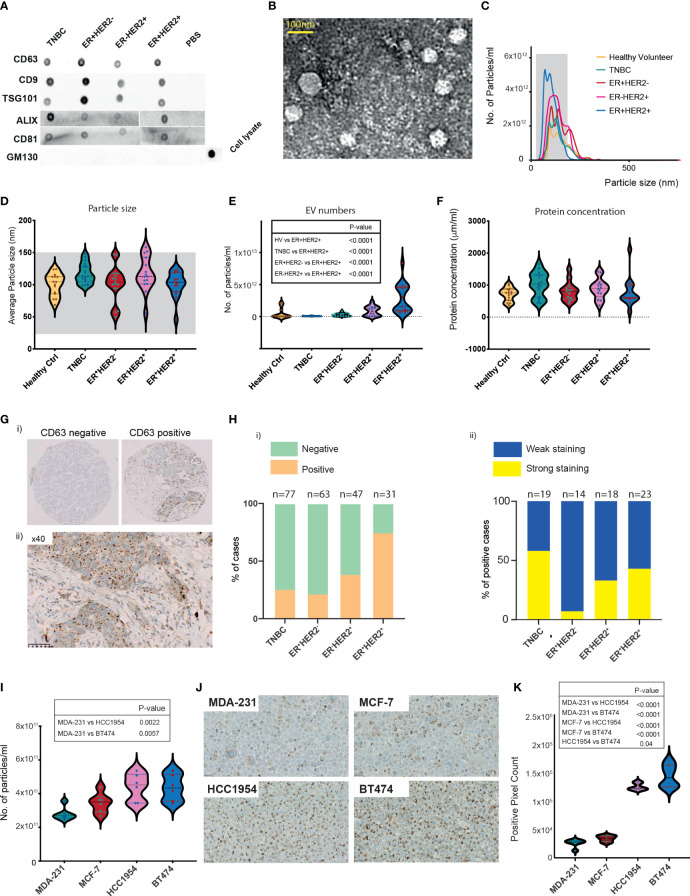
Characterisation of breast cancer patient serum- and cell-line-derived EVs. **(A)** Representative dot blots of the isolated EV fraction. Common EV markers (CD63, CD9, TSG101, ALIX, CD81) and Golgi GM130 markers were used for characterisation. **(B)** Representative image of homogeneous, intact, round vesicles observed by transmission electron microscopy. **(C)** Nanosight tracking analysis (NTA) of EVs derived from sera of HV and breast cancer subtypes. The grey-shaded area defines the expected particle size of exosomes (30–150 nm in diameter). **(D–F)** Violin plots of **(D)** size (mean HV=100.3 nm, TNBC = 117.6 nm, ER^+^HER2^−^ = 101.8 nm, ER^−^HER2^+^ = 117 nm, ER^+^HER2^+^ = 100.7 nm). **(E)** Particle distribution (particles/ml) and **(F)** protein concentration of serum-derived EVs is shown. HV *n* = 15, TNBC *n* = 18, ER^+^HER2^−^ *n* = 15, ER^+^HER2^−^ *n* = 15 patient samples. Significant *p*-values (one-way ANOVA) are shown. **(G)** Representative images of CD63 immunohistochemical staining of a breast cancer tissue microarray are shown. **(H)** Bar chart illustrating **(i)** the percentage of CD63-positive and CD63-negative cases and **(ii)** the intensity of CD63 staining in breast cancer subtypes. **(I)** Violin plots of particle distribution (particles/ml) of breast cancer cell line-derived EVs are shown (*n* = 6 per group). Significant *p*-values (one-way ANOVA) are shown. **(J)** Representative images of CD63 immunohistochemical staining of breast cancer cell line pellets. **(K)** Violin plots of the CD63^+^ pixel count across differing breast cancer cell line pellets (*n* = 6 fields per section). Significant *p*-values (one-way ANOVA) are shown.

To investigate the relationship between BC patient-derived EVs and immune cell crosstalk, we prospectively recruited a total of 63 newly diagnosed breast cancer patients and 15 healthy volunteers (HV). Clinical characteristics are provided in **Table 1**. Following ultracentrifugation of serum collected prior to starting any anti-cancer treatments (defined as timepoint 1 (TP1)), EVs isolated were subjected to characterisation using three different methods. The sorting of cargo into exosomes involves specific proteins associated with the endosomal sorting complex required for transport (ESCRT), such as ALG-2-interacting protein X (ALIX) and tumour susceptibility gene 101 protein (TSG101) (17). Proteomic analysis has confirmed the enrichment of tetraspanin proteins CD9, CD81, and CD63 on exosomes (18). First, we confirmed that our EV preparations were positive for endosome-specific proteins, such as CD63, CD9, TSG101, ALIX, and CD81, but were negative for a *cis*-Golgi protein marker (GM130), which is absent in exosomes (**Figure 1A**). Transmission electron microscopy revealed vesicular structures comparable to previously described EVs (19–21) (**Figure 1B**). The size distribution and concentration (particles/ml) of the EVs were determined by nanoparticle tracking analysis (NTA) (**Figure 1C**). The mode for each sample from different groups fell within the exosomal size range (30–150 nm). However, the mean particle size for all groups classified these EVs as being primarily of the larger exosome vesicle subset, with no significant differences observed across different breast cancer groups (**Figure 1D**). Sera of patients with ER^+^HER2^+^ demonstrated a significantly higher number of EV/ml compared to HV and other BC subgroups (**Figure 1E**). Irrespective of this difference in particle concentration, the total protein concentration was similar across all groups (**Figure 1F**).

**Table 1:** Clinical characteristics of the study population.

	TNBC	ER^+^HER2^−^	ER^-^HER2^+^	ER^+^HER2^+^	Healthy volunteers
Numbers	18	15	15	15	15
**Age at diagnosis (median, range)**	51 (32–68)	50 (33–75)	53 (38–81)	44 (23–64)	38 (20–58)
Sex					
Female	100% (18)	100% (15)	100% (15)	100% (15)	100% (15)
Ethnicity					
Caucasian	50% (9)	67% (10)	60% (9)	73% (11)	60% (9)
BAME	27% (5)	20% (3)	33% (5)	27% (4)	40% (6)
Unknown	22 (4)	13% (2)	7% (1)	–	–
Histological type					
Ductal	94% (17)	80% (12)	73% (11)	87% (13)	NA
Lobular	6% (1)	20% (3)	4% (4)	13% (2)	NA
Grade (% *n*)					
1	6% (1)	7% (1)	–	–	NA
2	17% (3)	47% (7)	27% (4)	53% (8)	NA
3	78% (14)	47% (7)	73% (11)	47% (7)	NA
Stage at the time of bleed point (% *n*)					
1	22% (4)	20% (3)	0% (0)	27% (4)	NA
2	33% (6)	40% (6)	40% (6)	40% (6)	NA
3	22% (4)	20% (3)	27% (4)	7% (1)	NA
4	22% (4)	20% (3)	33% (5)	27% (4)	NA

NA, not applicable.

Neoadjuvant chemotherapy was started for 19/63 of the early BC patients shortly after study recruitment. These patients were then followed up with longitudinal blood draws at 3 weeks following the first cycle of chemotherapy (time-point 2 (TP2)), 3 weeks following the final cycle of chemotherapy (TP3), and 4 weeks following their definitive surgical management (TP4). The majority of these patients were TNBC cancer. We investigated the change in EV numbers in the sera of patients undergoing neoadjuvant chemotherapy (**Supplementary Figure S1A**). In line with König et al., we observed a statistically significant increase in the number of EVs/ml on chemotherapy (TP1 vs. TP2 *p* = 0.004; TP1 vs. TP3 *p* = 0.034) (**Supplementary Figure S1A**). This rise was short-lived following definitive breast surgical management, with a significant drop observed in the EV numbers at TP4 (TP3 vs. TP4 *p* = 0.009) (**Supplementary Figure S1A**).

Current evidence suggests that exosomes can fuse with the plasma membrane of the recipient cell and release their contents into the target cell (22). Given that the EV marker expression of CD63 in tumour tissues using immunohistochemistry has been shown to be feasible (23), we examined the expression patterns of CD63 staining in a tissue microarray (TMA) of early BC patients (*n* = 218) (**Figure 1G**). Cases were scored positive when any degree of multivesicular granule staining was observed (**Figure 1H**(i)) and further classified as demonstrating weak or strong staining (**Figure 1H**(ii)). CD63 staining was identified in 25% of TNBC (19/77), 22% (14/63) of ER^+^HER2^−^, 38% (18/47) of ER^−^HER2^+^, 74% (23/31) ER^+^HER2^+^ breast cancer cases (**Figure 1H**(i); **Supplementary Figure S1B**) (Chi-square *p* < 0.0001), possibly helping to hypothesise that the observed significantly higher number of EVs in sera of ER^+^HER2^+^ BC patients may reflect a higher number of tumour-derived EVs. Interestingly, of the positive cases, TNBC cancers represented the highest proportion of the cases with strong CD63 staining intensity (**Figure 1H**(ii)), with 58% of the positive cases demonstrating strong CD63 immunohistochemistry (IHC) expression compared to only 7% of ER^+^HER2^−^ cases.

For further validation, we isolated EVs from MDA-231, MCF-7, HCC1954, and BT474 human breast cancer cell lines. The morphology of the EV preparations was visualised by electron microscopy, as illustrated in **Supplementary Figure S1C**. All samples appeared as intact round vesicles without debris or aggregates. The vesicles were positive for the EV tetraspanin marker CD63 but negative for the *cis*-Golgi protein marker GM130 (**Supplementary Figure S1D**). The majority of the vesicles ranged in size from 30 to 150 nm for all four cell lines (**Supplementary Figure S1E**). However, distribution analysis revealed a subtype-specific difference in the mean size of the vesicles, with EVs from the TNBC cell line, MDA-231 significantly smaller than MCF-7, HCC1954, and BT474 cell-line-derived EVs (**Supplementary Figures S1E, F**). In keeping with the patient serum data, HER2^+^ cell lines (HCC1954 and BT474) were observed to have significantly higher numbers of EVs in the culture media (**Figure 1I**). Expression of CD63 in formalin-fixed, paraffin-embedded (FFPE) BC cell-line pellets also supported this observation (**Figures 1J, K**), with HER2^+^ breast cancer cell lines demonstrating significantly higher levels of CD63 staining (confined often to discrete intracellular structures) than non-HER2^+^ (MDA-231 and MCF7) cell-line pellets (**Figure 1K**).

### Differential immune cell composition across high and low extracellular vesicle-associated signature scores

**Figure 2:**
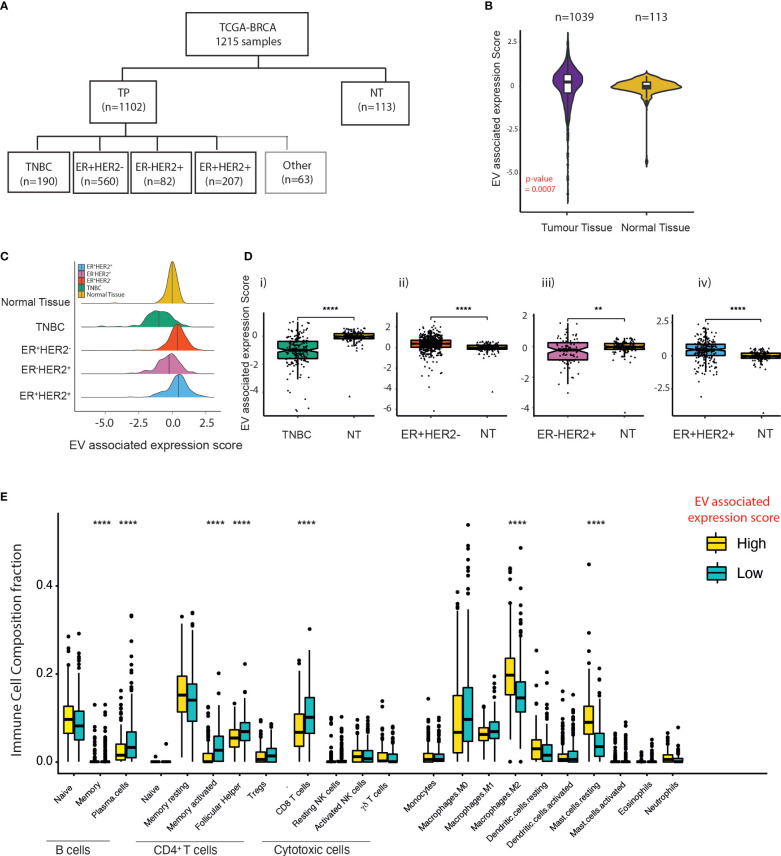
EV expression in breast cancer subtypes within the TCGA dataset. **(A)** Classification of breast cancer tumours according to the expression of ER and HER2 from TCGA breast cancer dataset. **(B)** Violin plots of the EV-associated expression scores in tumour samples (*n* = 1,039) compared to adjacent normal mammary tissue (*n* = 113). The Kruskal–Wallis test was used for comparison. **(C)** Density plot of the distribution of EV-associated expression scores across TNBC, ER^+^HER2^−^, ER^-^HER2^+^, and ER^+^HER2^+^ breast cancer subtypes and “normal (adjacent breast) tissue” from TCGA database. **(D)** The EV expression score of tumour tissue compared to adjacent normal mammary tissue across differing breast cancer subtypes is shown. The median and quantiles are shown. The Kruskal–Wallis test was used for comparison (^****^ *p* < 0.0001; ^**^ *p* < 0.01). **(E)** Immune cell infiltration scores for the EV-high and EV-low tumours determined by CIBERSORT. The median and quantiles are shown. Wilcoxon signed-rank test was used for comparison (^****^ *p* ≤ 0.0001).

Next, to understand the impact of EV secretion and its link to immune cell composition across different BC subtypes, we extracted BC data from The Cancer Genome Atlas (TCGA). In total, 1,215 transcriptomes were drawn (TNBC = 190, ER^+^HER2^−^ = 560, ER^-^HER2^+^ = 82, ER^+^HER2^+^ = 207, and normal mammary tissues = 113) (**Figure 2A**). Using an in-house bioinformatics algorithm, the expression signatures of five known essential EV markers (CD63, CD9, CD81, TSG101, and Alix) were utilised for the computation of EV-associated expression scores. EV-associated expression of tumour tissues (*n* = 1,039) was higher than that found in normal tissues (*n* = 113) (*p*-value = 0.0007; **Figure 2B**); this was validated further in data from paired tumour and adjacent normal tissues (**Supplementary Figure S2A**). There were significant subtype-specific differences in the EV-associated expression scores across molecular subtypes of BC (**Figure 2C**). The EV-associated expression for TNBC and ER^-^HER2^+^ BCs was lower than that of ER^+^ BCs (**Supplementary Figure S2B**). Specifically, TNBC BCs demonstrated the lowest EV-associated expression scores compared to other BC subtypes (**Figure 2C**; **Supplementary Figure S2B**). Interestingly, we observed that for ER^-^ (TNBC and ER^-^HER2^+^) BCs, the EV expression score in the tumour tissue was lower than the normal adjacent mammary tissue (**Figure 2D**(i, iii)); in contrast to ER^+^ BCs (**Figure 2D**(ii, iv)).

To examine the relationship between EV-associated expression and immune cell infiltration, 30 cases in the TCGA-BC cohort were identified at random for TIL evaluation of H&E images (**Supplementary Figure S2C**). Median TIL levels were 10% (range 1%–90%; the first and fourth quartiles were 5% and 31.25%) with a heterogeneous distribution across the samples analysed (**Supplementary Figure S2C**(i, ii)). Despite a low number of cases analysed, we observed a statistically significant negative correlation between EV-associated expression scores and stromal TIL counts (Pearson *r* = 0.57; *p* = 0.0005) (**Supplementary Figure S2D**). CIBERSORT analysis on the TPM gene expression of BCs identified that samples defined as having low EV-associated expression scores had a significantly higher abundance of plasma and memory B cells, CD4^+^ memory-activated T cells, T follicular cells, and CD8 T cells (*p*-values < 0.0001). Samples with high EV-associated expression scores were more enriched in anti-inflammatory M2 macrophages and mast cells (**Figure 2E**), providing evidence that EVs within cancers may help to shape the immune TME within BCs.

### Extracellular vesicles from TNBC cancers promote the most suppressive phenotype in CD3^+^ T-cell *in vitro*

**Figure 3:**
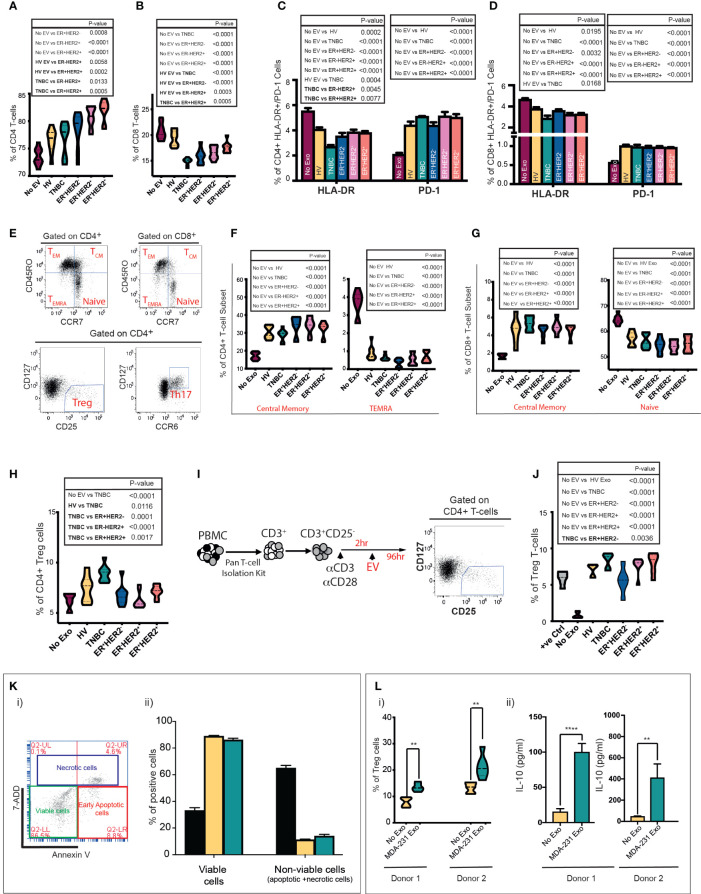
Serum and cell-line-derived EVs promote a suppressive phenotype in T cells. Healthy volunteer peripheral blood mononuclear cells (PBMCs) were cultured *in vitro* unstimulated or stimulated with EVs from HV (*n* = 9) or TNBC, ER^+^HER2^−^, ER-HER2^+^, or ER^+^HER2^+^ patients for 4 days (*n* = 36; nine per each breast cancer subtype). **(A, B)** Pooled data (*n* = 9 per cohort) of the percentages of **(A)** CD4^+^ T-cell and **(B)** CD8^+^ T-cell lymphocyte subsets detected by flow cytometry following co-cultures are shown. Significant *p*-values (one-way ANOVA) are shown. F(DFn, DFd): **(A)** *F*(5, 48) = 14.40 and **(B)** *F*(5, 48) = 26.48. **(C, D)** Percentages of HLA-DR and PD-1 expression on **(C)** CD4^+^ and **(D)** CD8^+^ T cells detected by flow cytometry are shown. Significant *p*-values (one-way ANOVA) are shown: F(DFn, DFd): CD4^+^HLA-DR^+^: *F*(5, 48) = 18.90 and CD4^+^PD-1^+^: *F*(5, 48) = 26.48; CD8^+^HLA-DR^+^: *F*(5, 48) = 10.91 and CD8^+^PD1^+^: *F*(5, 48) = 15.80. **(E)** Representative dot plots showing the different T-cell subpopulations defined by flow cytometry analysis are shown. PBMCs were separated into T-cell subsets: Treg and Th17, and based on the differential surface expression of CD45RO and CCR7; the relative percentage of naïve, central memory, effector memory, and terminally differentiated effector memory cells (TEMRA) CD4+/CD8+ T cells was analysed. **(F, G)** Frequency of central memory and TEMRA CD4^+^ **(F)** and central memory and naive CD8^+^ T-cell subsets **(G)** between groups are shown. Significant *p*-values (one-way ANOVA) are shown. F(DFn, DFd): CD4^+^CM: *F*(5, 48) = 24.55; CD4^+^TEMRA^+^: *F*(5, 48) = 96.71; CD8^+^CM: *F*(5, 48) = 23.56 and CD8^+^naive^+^ *F*(5, 48) = 21.65. **(H)** Flow cytometry analysis of the frequency of Tregs following PBMC stimulation. Significant *p*-values (one-way ANOVA) are shown. F(DFn, DFd): *F*(5, 48) = 11.05. **(I)** Schematic representation of the experimental approach for isolation and stimulation of CD3^+^CD25^−^ naïve T cells. Following the isolation of CD3^+^/CD25^−^ naïve T cells from PBMCs using a Pan T-cell isolation kit, naïve cells were seeded in 96-well U-bottom plates and stimulated with CD3/CD28 activation beads plus 0.2 ng/ml IL-2. Following 2 h of incubation time, the same number of serum-derived EVs were added per well. Cells were cultured for 96 h prior to analysis by flow cytometry. **(J)** Quantification data of the percentage of Tregs following CD3^+^CD25^−^ T-cell stimulation. Anti-CD3/CD28-coated Dynabeads were used as a positive control with maximum-level polyclonal TCR activation. Significant *p*-values (one-way ANOVA) are shown. F(DFn, DFd): *F*(6, 35) = 30.41. The plots shown are pooled data from three independent experiments (except for plot J which represents pooled data from two independent experiments). **(K)(i)** Representative plot of annexin V and 7-AAD staining by flow cytometry. Live cells are negative for annexin-V and 7AAD. The total nonviable cell count was calculated by adding a total of 7AAD (necrotic) and annexin V-positive (apoptotic) cells. Experiments were performed in triplicate wells for each condition. **(K)(ii)** Bar graph representing the mean proportion of live and nonviable cells analysed in **(i)**. Positive control: an aliquot of unstimulated PBMCs heated to 65°C for 10 min, chilled on ice, and mixed with an aliquot of live unstimulated PBMC. Significant *p*-values (two-way ANOVA) are shown: F(DFn, DFd): *F*(4, 20) = 37.09). **(L)(i)** Tregs after MDA-231-derived EV stimulation in the two donors are shown. Significant *p*-values (Mann–Whitney *t*-test) are shown. **(L)(ii)** IL-10 quantification in supernatant from EV-PBMC co-cultures from both donors 1 and 2 is seen. All experiments were performed in triplicate wells for each condition and repeated at least twice. Pooled data are shown. Significant *p*-values (Mann–Whitney *t*-test) are shown (donor 1 *p* < 0.001 and donor 2 *p* = 0.0023).

Incubation of immune cells with tumour cell-derived EVs can bias them toward an anti- or proinflammatory response (24, 25). We sought to determine if circulatory EVs influence the inflammatory response in T cells in a subtype-specific manner. *In vitro* stimulation of peripheral blood mononuclear cells (PBMCs) with EVs from HV, TNBC, ER^+^HER2^−^, ER^-^HER2^+^, and ER^+^HER2^+^ groups demonstrated a general trend for an increase in the CD4^+^ T cell (**Figure 3A**) and a statistically significant decrease in CD8^+^ T cell (**Figure 3B**) populations of PBMCs when co-cultured with BC-derived serum EVs as compared to nonstimulated PBMCs. The higher frequency of CD4^+^ T cells observed was only statistically significant across non-TNBC subgroups, and these groups also demonstrated statistically significant differences as compared to HV or TNBC-derived EV stimulation conditions. We observed that the decrease in CD8^+^ T cells was most profound when cultured with TNBC-serum-derived EVs (**Figure 3B**).

Next, we investigated the change in expression of the human leukocyte antigen DR (HLA-DR), a well-recognised marker of T-cell activation (26, 27), and programmed cell death-1 (PD-1) as a marker associated with T-cell suppression. The cellular phenotypes of CD3^+^CD4^+^HLA-DR^+^/CD3^+^CD8^+^HLA-DR^+^ and CD3^+^CD4^+^PD-1^+^/CD3^+^CD8^+^PD-1^+^ T cells were characterised by flow cytometry (**Figures 3C, D**). The presence of circulating serum-derived EVs from healthy volunteers and BC patients *in vitro* resulted in a decrease in the frequency of CD3^+^CD4^+^HLA-DR^+^ (**Figure 3C**) and CD3^+^CD8^+^HLA-DR^+^ T cells (**Figure 3D**). This effect was most profound following co-culture with TNBC EVs across both T-cell subsets; with a statistically significant difference also observed between TNBC and ER^+^HER2^+^ conditions for CD3^+^CD4^+^HLA-DR^+^ T cells and between HV and TNBC conditions for CD3^+^CD8^+^HLA-DR^+^ T cells. In contrast, we observed that co-culture with EVs derived from HV and all 4 types of BC resulted in a higher percentage of CD3^+^CD4^+^PD-1^+^ (**Figure 3C**) or CD3^+^CD8^+^PD-1^+^ (**Figure 3D**) T cells. No statistically significant differences were observed between EV-stimulated groups.

Next, we examined T-cell subsets in detail, specifically CD4^+^CD127^−^CD25^hi^ T regulatory cells (Tregs), CD4^+^CD127^hi^CCR6^hi^ Th17 cells, and we used differentiation markers CD45RO and CCR7 to subdivide CD8^+^ and CD4^+^ T cells into naïve (CD45RO^-^CCR7^+^), central memory (CD45RO^+^CCR7^+^; T_CM_), effector memory (CD45RO^+^CCR7^−^; T_EM_), and terminally differentiated effector memory cells re‐expressing CD45RA (CD45RO^−^CCR7^−^; T_EMRA_) (**Figure 3E**) (28). PBMCs cultured with serum-derived EVs from HV and all four BC types had a higher percentage of CD4^+^T_CM_ and CD8^+^T_CM_ T cells than the unstimulated PBMCs (**Figures 3F, G**, respectively). Inversely, the percentages of CD4^+^T_EMRA_ (**Figure 3F**) and naïve CD8^+^ (**Figure 3G**) T cells were lower in the serum-derived EV-stimulated conditions. No statistically significant changes in the CD4^+^ naïve, T_EM_ or Th17 cells were observed (data not shown). We observed that PBMC cultured with TNBC serum-derived EVs resulted in a significantly higher frequency of induced Tregs *in vitro* compared to all other experimental conditions (**Figure 3H**). To investigate this effect further, naïve CD4^+^CD25^−^ T cells activated with CD3/CD28 beads followed by co-culture with HV, TNBC, ER^+^HER2^−^, ER^-^HER2^+^, and ER^+^HER2^+^ EVs (**Figure 3I**) were examined. We observed an increased percentage of Tregs in culture compared to the activated T cells without EV stimulation (**Figure 3J**). This increase in the frequency of induced Tregs was largest if stimulated with EVs derived from the serum of TNBC cancer patients.

We have previously shown that longitudinal monitoring of tumour immune dynamics, especially in TNBCs, during chemotherapy may help to predict therapeutic response early (29). Chen et al. (30), reported that in patients with metastatic melanoma, the level of circulating exosomal PD-L1 changes during anticancer therapy and correlates with response. We investigated if there was a relationship between exosomal PD-L1 expression and chemotherapy response in TNBC patients receiving neoadjuvant chemotherapy (see **Supplementary Figure S1A**). Although not statistically significant, we observed a trend for higher exosomal PD-L1 expression in “responder” patients as compared to those who demonstrated “resistant” disease approximately 4 weeks following their definitive surgical management (TP4) (**Supplementary Figure S3A**). These data are in line with our previous work in preclinical models of TNBCs (31) showing that PD-L1 is secreted on exosomes in an ALIX (a critical mediator of exosome biogenesis)-dependent manner and impaired exosomal release confers an enhanced immunosuppressive phenotype on tumour cells.

To further investigate if the immunomodulatory effects observed, especially with TNBC serum-derived exosomes, are driven by the tumour-cell-derived EVs, we isolated EVs from MDA-MB-231 (TNBC) human breast cancer cell lines and characterised them as shown in **Supplementary Figures S1C–F**. Prior to studying the immunoregulatory effects of tumour-derived EVs on PBMCs in our co-culture assay, we determined if the EVs directly induced apoptosis/necrosis of immune cells by assessment of annexin V and 7-AAD staining by flow cytometry. The cells were identified as necrotic/late apoptotic (7AAD+ annexin V+), early apoptotic (annexin V high and 7AAD negative), and live cells (7AAD and annexin V negative) (**Figure 3K**(i)). The sum of necrotic and apoptotic cells constituted the total percentage of dead cells. Following incubation of healthy donor PBMCs with breast cancer cell-line EVs, we did not observe a significant decrease in the percentage of viable cells following co-culture or an increase in the percentage of apoptotic/necrotic cells in PBMCs exposed to MDA-231 cell-line-derived exosomes (**Figure 3K**(ii)). We cultured healthy volunteer PBMCs from two donors with TNBC MDA-231-derived EVs (**Figure 3L**). We confirmed that MDA-231 cell-line-derived EVs increased the percentage of Tregs cells in both co-culture conditions (**Figure 3L**(i)), with an associated increase in IL-10 levels in the supernatant of MDA-231 EV-stimulated PBMC co-cultures (**Figure 3L**(ii)), providing further support to our earlier observations that serum-derived TNBC exosomes can induce an immunosuppressive response.

### Differential cytokine production of PBMC cultures stimulated with TNBC cell-line derived extracellular vesicles

**Figure 4:**
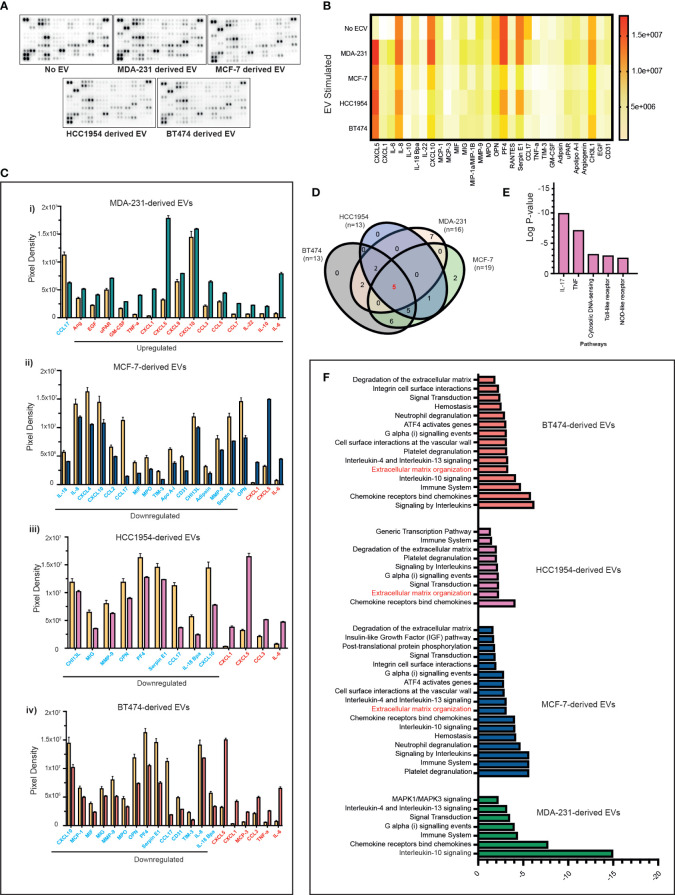
Proteome profiles of secreted factors from nonstimulated and BC cell line EV-stimulated healthy volunteer PBMCs. **(A)** Representative human XL cytokine array dot blots are shown. **(B)** A heatmap of the analysis of dot blot intensity across all experimental conditions is shown. **(C)** Bar chart showing significant changes in analytes detected in PBMC culture supernatants stimulated with a cell line-derived EVs: **(i)** MDA-MB-231, **(ii)** MCF-7, **(iii)** HCC1954, and **(iv)** BT474-derived EVs compared to unstimulated PBMC. Upregulated analytes are illustrated in red and those downregulated are shown in blue. **(D)** The Venn diagram is used to identify overlapping and nonoverlapping up- or downregulated cytokines in the analysis of nonstimulated compared to MDA-231, MCF-7, HCC1954, and BT474 EV stimulated. **(E)** REACTOME pathway analysis of the commonly altered cytokines in EV-stimulated immune cells compared to nonstimulated immune cells. **(F)** Pathway analysis of specific analytes up- or downregulated in the supernatant of PBMC co-cultures with MDA-231, MCF-7, HCC1954, and BT474 EVs.

Next, we investigated if the above-mentioned changes in the immune cells induced by EVs were also associated with changes in the cytokine profile. However, serum-derived EVs are a heterogeneous population originating from blood cells, endothelial cells, and cancer cells, so we focussed our experiments primarily on PBMC and tumour cell-line-derived EV co-cultures. We stimulated PBMCs with differing breast cancer subtypes and analysed the cytokine modulation triggered by the exosomes in the supernatants following co-culture experiments. Supernatants were analysed for 105 different cytokines, chemokines, and growth factors using a cytokine array (**Figure 4A**). In total, 30 analytes were observed to be significantly differentially expressed in the culture media compared to nonstimulated EV conditions (**Figure 4B**). The majority of the analytes were significantly upregulated in the supernatant of PBMCs stimulated with MDA-231-derived EVs (**Figure 4C**(i); upregulated analytes marked in red and downregulated analytes marked in blue) as compared to MCF-7, HCC1954, and BT474-derived EVs (**Figure 4C**(ii–iv)). Only changes in five analytes (CXCL5, CXCL1, CCL17, CXCL10, and IL-6) were seen to overlap across all four breast cancer cell-line EV-stimulated conditions (**Figure 4D**). Except for CXCL10, the direction of change of these analytes (CXCL5, CXCL1, CCL17, and IL-6) was consistent across all groups. On the contrary, CXCL10 was significantly upregulated in the supernatant of MDA-231 EV-stimulated co-culture but downregulated in MCF-7, HCC1954, and BT474 EV-stimulated assays. REACTOME pathway analysis identified these overlapping analytes as highly enriched in the IL-17 and TNF-α pathways (**Figure 4E**; p-2.28e^−08^ and 7.43e^−06^, respectively). Interestingly, pathway analysis of specific analytes upregulated in the supernatant of PBMC co-cultures with MDA-231 EVs observed an enrichment of the IL-10 signalling pathways, in keeping with the increase in Treg populations observed previously (**Figure 4F**). In contrast, pathway analysis of significantly differential analytes in the supernatant of PBMC co-cultures with MCF-7, HCC1954, and BT474 EVs identified extracellular matrix remodelling as an important pathway altered across all groups (**Figure 4F**), possibly providing a mechanism for the observed subtype-specific organotropism for metastasis development in breast cancers (32).

## Discussion

Recent evidence points to sophisticated intercellular communication between cancer cells and the host environment through secreting EVs (33, 34), and profiling of breast cancer cell-line-derived EVs has demonstrated that EV content is diverse in nature and varies depending on the parent cell (35). Here, we observed clear subtype-specific differences in the EVs. For example, TNBC (serum or cell line) produced the smallest number of EVs, whilst the highest concentration of EVs was detected in ER^+^HER2^+^ cancers, reflecting the higher level of CD63^+^ vesicles found within its local tumour microenvironment. HER2^+^ EVs have been shown to be enriched for tumour cell proliferation proteins (36) and their release has been shown to be modulated by growth factors in the surrounding microenvironment such as EGF and heregulin, two of the known HER2 receptor-activating ligands (36), providing some insights into the observed differences. The downstream effect of this diversity on EV immune regulation within breast cancer subtypes is less clear. To the best of our knowledge, this is the first study to directly investigate the differing immunomodulatory functions of patient serum-derived and cancer cell-line-derived EVs across different breast cancer subtypes.

Breast cancer subtypes differ in the level of immune infiltration observed in the tumour. Comparatively, more patients with TNBC fall into the category of having a better T-cell infiltrate than any other subtype. ER-positive disease in contrast is associated with the least immune infiltrates. We observed that the EV-associated expression of breast tumour tissues in the TCGA dataset of ER-negative disease (TNBC and ER^-^HER2^+^) was lower than that found in adjacent normal tissues. This along with the observation that low EV-associated expression scores in cancers were associated with a higher abundance of several proinflammatory immune cell infiltrates; highlights the need for further investigations into how EVs can influence/be influenced by the local immune tumour microenvironment.

The specific recognition of cognate antigenic peptides presented by MHC molecules triggers T-cell receptor (TCR) signalling, determining T-cell fate and function. Cancer-derived EVs have been shown to carry MHC class I and II antigens (37, 38), and membrane-associated death ligands such as FasL or TRAIL (39). We observed a shift in the T-cell population from naïve to central memory CD4^+^ and CD8^+^ T cells following stimulation of serum-derived EVs (in healthy and cancer patients), providing evidence of initial activation of T cells following EV antigen presentation *in vitro.* Cancer-derived EVs have been reported to escape immune surveillance by mechanisms that are numerous and varied (40–43). We report that EVs from patients diagnosed with TNBCs promote the most suppressive phenotype in CD3^+^ T cells *in vitro*, resulting in a decrease in activated CD8^+^ T cells whilst increasing suppressive cells such as Tregs. TNBC-derived EVs being the front runners for their propensity to regulate immune surveillance mechanisms compared to other breast cancer subtypes is perhaps not that surprising, given their recognition as the more immunogenic breast cancer subtype with a higher degree of stromal and intratumoural TIL interactions (44). Similarly, PD-L1 expression is reported to be significantly higher in TNBC cancers compared to non-TNBC cancers (45), and MDA-231 breast cancer cells have been shown to release EVs carrying PD-L1. Vesicular PD-L1 functions to suppress T cell killing through direct binding with PD-1 on T cells, causing inhibition of CD3/CD28-induced ERK phosphorylation and NF_k_B activation on T cells (46, 47). We observed a trend for lower exosomal PD-L1 expression in TNBC patients with poor prognosis. These findings are in line with our previous findings that ALIX-depleted cells exhibit increased surface levels of PD-L1, conferring an enhanced immunosuppressive phenotype on breast cancer cells (31). Exosomes derived from highly metastatic breast cancer cells have also been shown to directly suppress T-cell proliferation and inhibit natural killer (NK) activity (48). These findings have important implications for understanding the underlying mechanisms of immunosuppression in breast cancer.

It would, however, be a missed opportunity to disregard the immunosuppressive effects of ER^+^HER2^−^-, ER^−^HER2^+^-, or ER^+^HER2^+^-derived EVs as not being as relevant as TNBC EVs. It is possible that EVs from different breast cancer subtypes have varying propensities to serve as trafficking vehicles to deliver PD-L1 into cells in the TME to modulate immune surveillance. An improved understanding of the levels and spatial relationship of EVs (e.g., suppressive PD-L1^+^CD63^+^ EVs vs. PD-L1^−^CD63^+^ EVs) to the tumour, immune, and stromal cells in the TME may pave the way for combining an EV secretion inhibitor and anti-PD-L1 therapy to improve anti-tumour response in all BC patient subgroups. Additionally, within the TME, IL-6 signalling has been linked to tumourigenesis in numerous mouse models and human cancers by driving tumour cell proliferation, protecting tumour cells from cell death, and promoting angiogenesis and metastasis (49–51). We observed upregulation of the IL-6 cytokine in the supernatant of EV-stimulated PBMCs across all four breast cancer subtypes. However, IL-6 is a complex pleiotropic cytokine and has also been shown to provide anti-tumour immunity by mobilising T-cell responses with broad effects on T-cell survival, proliferation, differentiation, and recruitment (52). Investigations on how EV-induced IL-6 upregulation within each breast cancer subtype TME may determine the tipping point between protumour and antitumour effects are warranted.

Cytokine profiling experiments identified a greater interaction of non-TNBC subtype EVs with signalling pathways involved in extracellular matrix organisation. Recently, integrin expression profiles of circulating EVs have been shown to direct organ-specific colonisation by fusing with cell-associated extracellular matrix (ECM), mediating EV uptake in specific target organs (32). The metastatic pattern of BCs varies by receptor status; TNBCs show an increased incidence of visceral and cerebral distant metastasis, while HR^+^ tumours have been shown to have a greater tendency to develop bone metastasis (36, 53, 54). Collectively, these data support further investigations into the subtype-specific EVs to decipher the mystery of distinct organotropism in breast cancers.

Our results could be extended to perform *in vitro* assays of patient-derived EVs titrated with immune cells isolated from the blood of the same patient to fully discern and measure immunological abnormalities induced by EVs. Research efforts to use EVs as biomarkers of tumour progression and/or immune suppression in cancer are technically challenging, given the “mix” of EVs present in circulation. Our data suggest that effects on immune cells from breast cancer patient-derived EVs could act as surrogate biomarkers of cancer-related immune deficiencies during cancer progression or cancer treatment following immunotherapies.

In conclusion, our study supports further investigations into how tumour-derived EVs are a mechanism that cancers can exploit to promote immune suppression and how breast cancer subtypes produce EVs with differing immunomodulatory capabilities. Understanding the intracellular/extracellular pathways implicated in the alteration from an active to a suppressed immune state by EVs is essential for developing effective EV-targeted treatments aimed at restoring the immune competence of a patient.

## Materials and methods

### Clinical material

Archived human serum samples from breast cancer patients (*n* = 63 (TNBC *n* = 18; ER^+^HER2^+^ *n* = 15, ER^−^HER2^+^ *n* = 15; ER^+^HER2^+^ *n* = 15)) and healthy volunteers (HV) (*n* = 15) were acquired following informed consent (BTBC study: REC No.: 13/LO/1248, IRAS ID 131133). This study received approval from King’s College London and GSTT Foundation NHS Trust research ethics committee and was conducted adhering to the principles of the Declaration of Helsinki. The samples were analysed in a double-blinded study where the clinicopathological reports of the sample were not revealed to the investigator until after the completion of the analysis.

### Purification of EVs

Serum samples were thawed at room temperature (RT), and 500 µl of human serum was diluted 1:1 using PBS and centrifuged at 12,200×*g* for 45 min. The collected supernatant was centrifuged at 100,000×*g* for 2 h at 4°C (Beckman Coulter, Indianapolis, USA, Optima™Max-XP) to pellet EVs. The pellet was washed in phosphate-buffered saline (PBS) and spun at 100,000×*g* for 1 h, before resuspending in PBS. Cell lines representative of four major types of breast cancer (MDA-MB-231 (termed MDA-231) TNBC, BT474 ER^+^HER2^+^, HCC1954 ER^−^HER2^+^, and MCF7 ER^+^HER2^−^) were cultured in RPMI 1640 Medium (Sigma-Aldrich, Gillingham, Dorset, UK) supplemented with 10% foetal bovine serum (FBS) and 100 µg/ml penicillin/streptomycin [Thermo Fisher Scientific (Life Technologies) Paisley, Scotland, UK]. Cells at approximately 80% confluency were washed with PBS and cultured in FBS-free media for the collection of EVs. After 24 h, the conditioned culture medium was centrifuged at 300×*g* for 10 min to remove cell debris. The supernatant was then centrifuged at 5,000×*g* for 20 min to remove apoptotic bodies, 12,200×*g* for 60 min to remove microvesicles and finally at 100,000×*g* for 2 h at 4°C to pellet EVs (Beckman Optima LE-80K). The pelleted EVs were suspended in PBS and collected by ultracentrifugation at 100,000 g for 1 h.

### Characterisation of the purified EVs

#### Nanosight tracking

The size and concentration of purified EVs were determined using the NanoSight LM10 with an LM14 thermo-regulated laser unit, constant injection flow, and equipped fast video capture and particle-tracking software from Malvern, UK. Data were analysed using NTA 3.0 software with standardised thresholding (55). Three technical readings per sample were performed.

#### Electron microscopy

Purified EVs suspended in PBS were dropped on carbon film supported by R2/2 Quantifoil grids, previously rendered hydrophilic by glow discharging in the air. The samples were then negatively stained using 2% uranyl acetate and imaged with a Tecnai Spirit electron microscope (FEI) operating at 120 kV.

#### Dot blot analyses

Briefly, a pre-wet nitrocellulose membrane immersed in Tris-buffered saline (TBS-T; 0.01M Tris-HCl at pH 7.5, 0.1 M NaCl, 0.05% (v/v) Tween-20) was loaded with 5 µl sample, standardised by particles/ml or 1 µg of cell lysate protein measured using the Pierce BCA Protein Assay Kit [Thermo Fisher Scientific (Life Technologies) Paisley, Scotland, UK] according to manufacturer’s instructions. Nonspecific sites were blocked by incubating the membrane in TBS+5% dried milk powder for 30 min. After washing twice in TBS-T, the membrane was incubated with primary antibody overnight at 4°C and secondary antibody for 1 h at RT, with TBS-T washes between each step. The membrane was developed using ECL reagents A and B and imaged using the Syngene GeneGnome imaging system and GeneSys software. A panel of antibodies used is shown in **Supplementary Table S1**.

### Immunohistochemical Analysis of CD63 expression on formalin-fixed paraffin embedded breast cancer tissue and cell lines 

CD63 immunohistochemical staining was performed on primary breast tumour tissue and the four BC cell lines. FFPE TMAs were constructed from patient tumour samples in triplicate, mainly from the periphery of the carcinoma and other representative areas of the invasive tumour. BC cell lines were harvested from confluent T150 tissue culture flasks, pelleted, and fixed in a 10% natural buffer of formalin for 20 h, followed by processing and embedding in paraffin blocks. Expression of CD63 was assessed on 3-μm-thick TMA and cell pellet sections. They were stained using an automated VENTANA (Roche Diagnostics Ltd, Burgess Hill, West Sussex, UK) platform with an ultra-view universal DAB Detection kit, followed by a haematoxylin counterstain. IHC was performed using Anti-CD63 (1:100) Rabbit Polyclonal HPA010088 (Atlas Antibodies, Bromma, Sweden) with CC2 tissue pretreatment for 64 min at 95°C. A blank control was used for any unspecific background staining detection.

Two pathologists independently scored the staining of CD63 on breast tumour TMAs, which was scored as negative when no granule staining or if faint diffuse cytoplasmic staining was observed and was scored positive when any degree of multivesicular granule staining was observed. QuPath v.0.1.2 image analysis was used to quantify the positive pixel count.

### The Cancer Genome Atlas tumour analysis

Transcriptomes were extracted from TCGA (https://portal.gdc.cancer.gov) breast invasive carcinoma cohort, which includes four molecular subtypes of breast cancer: TNBC, ER^+^HER2^−^, ER^-^HER2^+^, and ER^+^HER2^+^, as well as normal mammary tissue adjacent to the tumour. A deconvolution model using five EV markers (CD63, CD9, CD81, TSG101, and ALIX) was developed to construct an EV signature score (see **Supplementary Methods**). A single-factor comparative method, the Kruskal–Wallis test, was used to examine the differences in EV-associated expression scores between different breast cancer subtypes and normal tissue samples.

To investigate the effect of EV-associated expression scores on immune cell populations, we examined the immune cell fractions of all samples using CIBERSORT (56) (see **Supplementary Methods**). In total, 1,000 iterations were performed to compute the relative proportions of 22 immune cell types using normalised gene expression data. For each sample, the confidence of deconvolution accuracy was estimated by a global *p*-value in the CIBERSORT, and only samples with a *p*-value of < 0.05 were used in subsequent analysis. To compare cell fractions in relation to the EV-associated expression scores, we computed quantiles of EV-associated expression scores across the samples. Samples were classified into high and low EV groups based on their EV-associated expression scores greater than 75% or less than 25% quantile. The Wilcoxon signed-rank test was used to compare the difference between immune cell types with high and low EV-associated expression scores. R 4.0.3 (https://www.R-project.org/) was used in all statistical analyses.

### Evaluation of tumour-infiltrating lymphocytes

TILs were evaluated for TCGA breast cohort, for which detailed biospecimen collection and processing protocols have been described elsewhere (57). The pathology team (PG) evaluated TILs based on the recommendations from the International Immuno-Oncology Biomarker Working Group on Breast Cancer (58). TILs were measured as the percentage of lymphocytes and macrophages within the total intratumoural stromal compartments.

### *In vitro* EV stimulation functional assays

Healthy donor PBMCs were isolated from whole blood using Ficoll–Paque density gradient centrifugation and plated at 2 × 10^5^ cells/well in 96-well U-bottom plates. Cells were cultured at 37°C with 5% CO_2_ for 4 days in the presence or absence of the same number of either serum-derived EVs from patients or cell line-derived EVs. The numbers added/well were based on the total number of EVs found in 20 µl of HV samples given, as these represented samples with the lowest concentration in most cases (median 7.5 × 10^8^. For each experiment, the same number of EVs were plated per well (range: 5.4 × 10^7^–4 × 10^9^) particles/well). Cells were then stained with a panel of antibodies (**Supplementary Table S2**). Experiments were performed in triplicate, and each well was analysed separately. Flow cytometry analysis was conducted on a BD LSR Fortessa flow cytometer. Data were analysed using the FlowJo software package (Tree Star, Ashland, OR, USA).

For assessment of stimulation of Tregs, naïve T cells were prepared from PBMCs using a Pan T-cell isolation kit (Miltenyi Biotech, Bisley, Surrey, UK 130-096-525) according to the manufacturer’s instructions. CD25^+^ cells were then depleted using CD25 microbeads (Miltenyi Biotec 130-092-983), and CD3^+^/CD25^-^ naïve T cells were then seeded in 96-well U-bottom plates at 2 × 10^5^ cells/well and stimulated with CD3/CD28 activation beads or CD3/CD28 Dynabeads at a ratio of 1 cell:1 bead plus 0.2 ng/ml IL-2. The same number of serum-derived EVs were added per well. As a positive control for Treg induction, control wells were stimulated with CD3/CD28 Dynabeads at a ratio of 1 cell:1 bead plus 5 nM retinoic acid, 0.2 ng/ml IL-2, and 10 ng/ml TGF-β. Cells were cultured at 37°C in 5% CO_2_ for 96 h and stained with a panel for Tregs prior to analysis by flow cytometry.

Cytokine profiles in culture media were assessed using the Proteome Profiler™ Array Human XL Cytokine Array Kit (ARY022B; Bio-Techne, Abingdon, UK) with analytes spotted in duplicate on each array. Imaging was performed using a Syngene GeneGnome imaging system, and Syngene Genetools software was used for analysis. IL-10 concentration was assayed using the Human IL-10 Quantikine ELISA Kit (D1000B; R&D Systems). Apoptosis was assessed using PE Annexin V Apoptosis Kit (BD Biosciences, Wokingham, UK 559763) according to the manufacturer’s instructions. Samples were analysed immediately using BD Accuri C6 Plus Flow Cytometer.

### Statistical analysis of functional assays

The means of all groups were compared for statistical differences by Student’s *t*-test or a one-way analysis of variance (ANOVA). A Bonferroni *t*-test was used, following the ANOVA, to understand the statistical difference between two groups when more than two groups were compared. Data are presented as means ± SEM. Significance levels were set to *p* < 0.05.

## Data availability statement

The original contributions presented in the study are included in the article/**Supplementary Material**. Further inquiries can be directed to the corresponding author.

## Ethics statement

The studies involving human participants were reviewed and approved by BTBC study: REC No: 13/LO/1248, IRAS ID 131133. The patients/participants provided their written informed consent to participate in this study.

## Author contributions

Conceptualisation and experimental design: SI, YZ, KA-J, TN and AT. Patient consent: SI, AT and TA. Additional sample provision: RM. Clinical data collection: TA. TCGA analysis: BZ and YZ. Experimental work: EV purification/*in vitro* studies/flow cytometry/cytokine analysis: RG, SS, AK and FF-B. Histopathology/immunohistochemistry/quantification: PG and IR. Nano sight tracking analysis: JV, AK and RL-O. Electron microscopy: FB. Manuscript preparation: SI, RG and PG. Figure preparation and statistical analysis: RG, AK and SI. All authors reviewed manuscript and provided intellectual content. Research supervised by and funding acquired by SI.
