# Supplementary figures and images for: Serum-derived extracellular vesicles from breast cancer patients contribute to differential regulation of T-cell-mediated immune-escape mechanisms in breast cancer subtypes
Source: Front Immunol. 2023 Jun 22;14:1204224. doi: 10.3389/fimmu.2023.1204224 (PMC10335744; doi:10.3389/fimmu.2023.1204224)

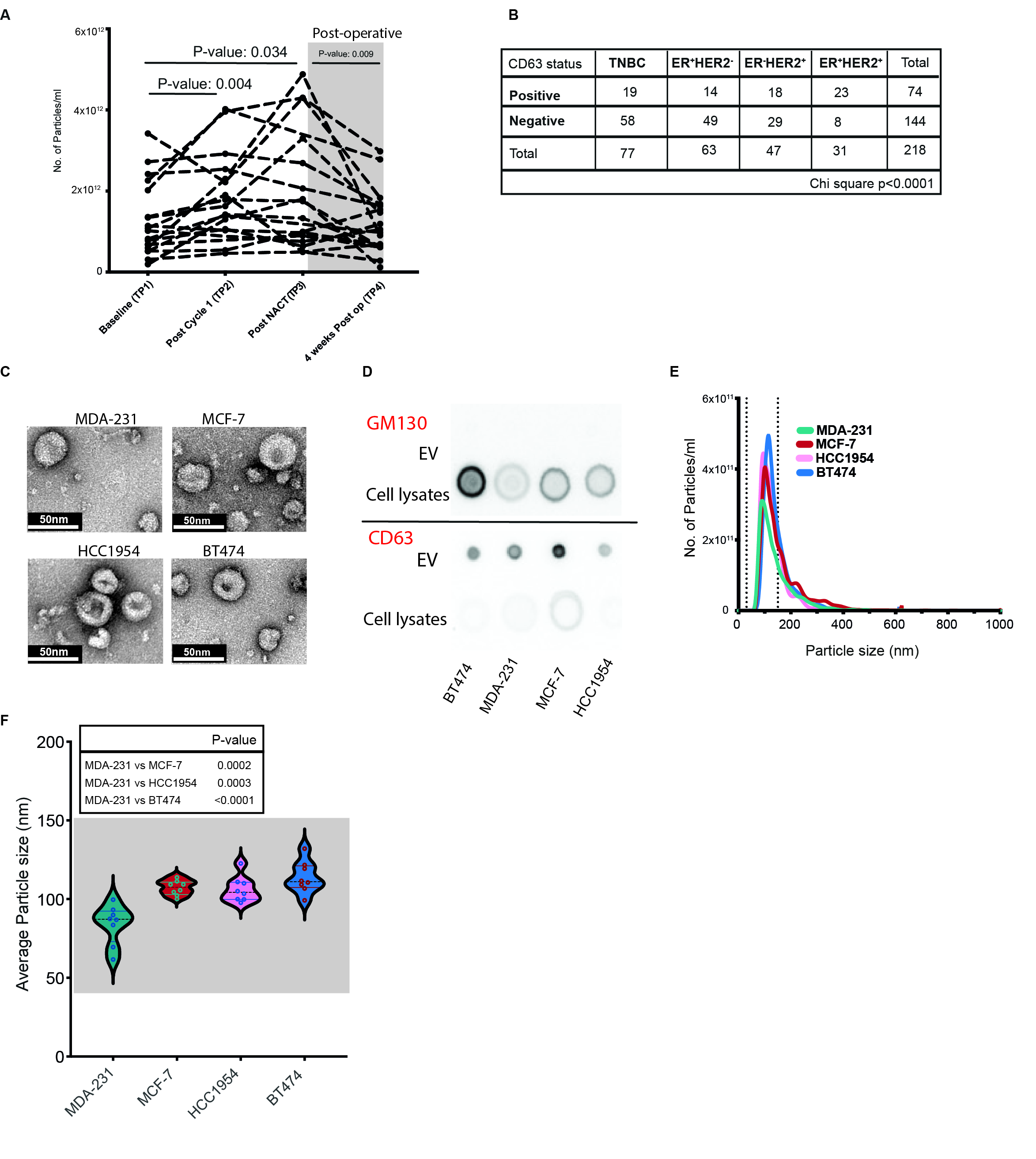

Supplement: Supplementary file 1 [file Image_1.tif]

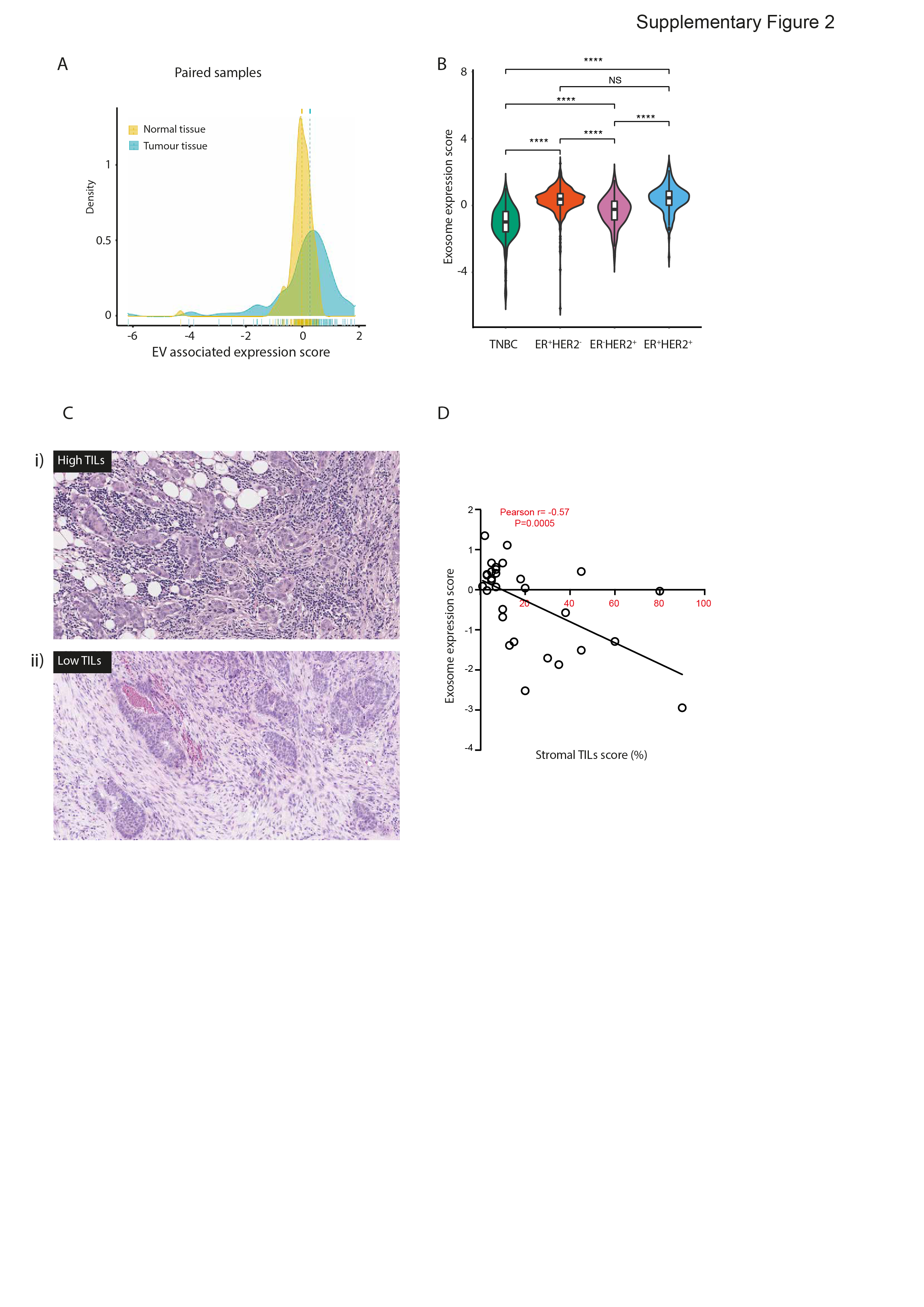

Supplement: Supplementary file 2 [file Image_2.tif]

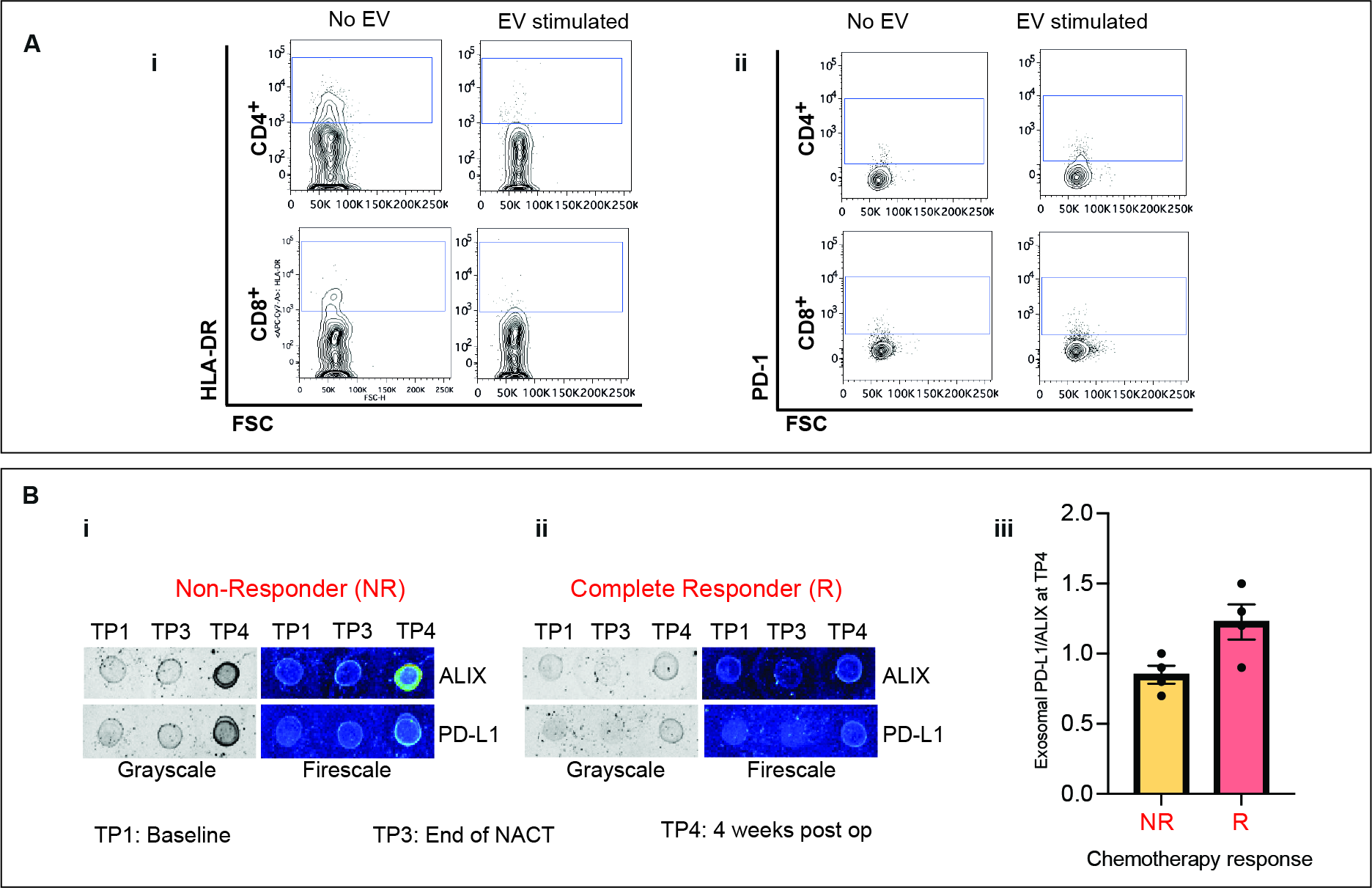

Supplement: Supplementary file 3 [file Image_3.tif]
