# Supplementary material for: Serum-derived extracellular vesicles from breast cancer patients contribute to differential regulation of T-cell-mediated immune-escape mechanisms in breast cancer subtypes
Source: Front Immunol. 2023 Jun 22;14:1204224. doi: 10.3389/fimmu.2023.1204224 (PMC10335744; doi:10.3389/fimmu.2023.1204224)
Supplement: Supplementary file 4 [file DataSheet_1.pdf]

Serum-derived extracellular vesicles from breast cancer patients contribute to differential regulation of T cell mediated immune-escape mechanisms in breast cancer subtypes.

Rosalind Graham[1#], Patrycja Gazinska[2,3#], Birong Zhang[4], Atousa Khiabany[1], Shubhankar Sinha[1], Thanussuyah Alaguthurai [1,2], Fabian Flores-Borja [5], Jose Vicencio[6], Fabienne Beuron[3], Ioannis Roxanis[3], Revadee Liam-Or[7], Andrew Tutt[2,3], Tony Ng[2,5,6], Khuloud Al-Jamal[7], You Zhou[4], Sheeba Irshad[1*]

[#]Joint first authorship

[*]*Corresponding author: email: sheeba.irshad@kcl.ac.uk*

1. **Supplementary Figure Legends**
2. **Supplementary Tables**
3. **Supplementary Methods**

Supplementary Figure Legends

Supplementary Figure 1: Characterization of breast cancer patient sera and cell-line derived EVs

A: Change in serum EV concentration (500μl of human serum) at various time points in breast cancer patients (n=19) undergoing neoadjuvant chemotherapy prior to surgery and 4 weeks following definitive surgery (shaded grey area). Wilcoxon matched pairs signed-rank test was used to detect significant differences in concentrations at different time points. **B:** Table illustrating CD63 expression, detected by IHC, in 218 breast cancer cases, broken down by subtype (Chi square P<0.0001). **C**: Representative images of homogeneous, intact, round vesicles observed by transmission electron microscopy for all 4 breast cancer cell lines. **D**: Dot blots of EV preparations and cell lysates from the 4 breast cancer cell lines using the EV marker CD63 and negative marker GM130 are shown. **E:** Nanosight tracking analysis of EVs from 4 breast cancer cell lines showing size distribution. Black vertical lines define the area expected for particles of exosomal size. **F:** Violin plots showing the average particle size of differing breast cancer cell line derived EVs (n=6 in each group). (Mean size 87.2, 107.5, 104.3, 111.1nm for MDA-231, MCF-7, HCC1954 and BT474 derived EVs, respectively). Significant P-values (one-way ANOVA) are shown.

Supplementary Figure 2: **EV associated expression in breast cancer subtypes within the TCGA dataset**

A: Density plot of the distribution of EV associated expression scores within tumour tissues and matched adjacent normal mammary tissue across breast cancer samples from TCGA database. **B**: Violin plots of the EV abundance in tumour samples across different breast cancer subtypes. Kruskal-Wallis test was used for comparison. **C**: Representative digitized H&E-stained whole-slide tumours from the TCGA defined as **i)** high TILs and **ii)** low TILs score. **D:** Scatter plot showing the Pearson correlation analysis between EV associated

expression scores and the tumour-infiltrating lymphocyte (TILs) scores of samples within TCGA (Pearson r=0.57; p=0.0005).

Supplementary Figure 3: Extracellular vesicles from TNBC cancers promote the most suppressive phenotype in $CD3^+$ T cells *in vitro.*

A: Gating strategy for (I) HLA-DR+ and (ii) PD-1 CD4+ and CD8+ T cells. **B**: ALIX and PD-L1 dot blots in (i) Non-Responders and (ii) Complete Responders during treatment. (**iii)** Bar chart representing PD-L1 and ALIX levels in responders versus non-responders after chemotherapy treatment.

Supplementary Tables

Table 1: Antibodies used for ECL/Dot Blot

Antibody	Conjugate	Clone	Catalogue	Company	Dilution
CD81	Unconjugated mouse IgG1	5A6	349502	Biolegend	1:1000
CD9	Unconjugated mouse IgG1	HI9a	312102	Biolegend	1:1000
CD63	Unconjugated rabbit	EPR5702	ab134045	Abcam	1:1000
Calnexin	Unconjugated rabbit IgG		10427-2-AP	Proteintech	1:1000
TSG101	Unconjugated rabbit IgG		14497-1-AP	Proteintech	1:1000
Calnexin	unconjugated Rabbit IgG	C5C9	2679T	Cell Signaling Technology	1:200
CD63	unconjugated	H5C6	NBP2-42225S	Novus Biologicals	1:200
GM130	Unconjugated IgG	EP892Y	AB52649	AbCam	1:1000
EpCAM	Unconjugated mouse IgG	VU1D9	SAB4700423	Sigma	1:200
Rabbit anti mouse IgG	HRP		7076S	Cell Signaling Technology	1:2000
Goat Anti-rabbit IgG	HRP		7074S	Cell Signaling Technology	1:2000

Table 2: Antibodies used for T cell characterisation by flow cytometry

Antibody	Conjugate	Clone	Catalogue	Company	Dilution
CD3	PerCP Cy5.5	UCHT1	560835	BD Pharmigen^{TM}	1:100
CD3	BV650	UCHT1	563851	BD Pharmigen^{TM}	1:100
CD4	BUV395	RPA-T4	564724	BD Horizon^{TM}	1:100
CD8	FITC	RPA-T8	561947	BD Pharmigen^{TM}	1:100
CD8	BV711	SK1	344734	Biolegend	1:100
CD127	Alexa Fluor 647	HIL-7R-M21	5650831	BD Pharmigen^{TM}	1:100
HLA-DR	APC-H7	G46-6	561398	BD Pharmigen^{TM}	1:100
CD45RO	AF700	UCHL1	561136	BD Pharmigen^{TM}	1:100
CD25	PE-CF594	M-A251	562403	BD Horizon^{TM}	1:100
CD196 (CCR6)	PE	11Ag	561019	BD Pharmigen^{TM}	1:100
CD183 (CXCR3)	PECy7	1C6	560831	BD Pharmigen^{TM}	1:100
PD-L1	Alexa Fluor 647	MIH5	566865	BD Pharmigen^{TM}	1:100
CD45RO	PE-Cy7	UCHL1	560608	BD Pharmigen^{TM}	1:100
CD197 (CCR7)	BV421	150503	562555	BD Horizon^{TM}	1:100
CD197 (CCR7)	AF700	G043H7	353434	Biolegend	1:100
PD-1	PE	MIH4	560908	BD Pharmingen^{TM}	1:100
PD-1	AF405	913429	FAB10861	R&D Systems	1:100
Fixable Viability Stain	FVS 510		564406	BD HorizonTM	1:500
Live Dead	Yellow		L34959	InVitrogen	1:1000

Supplementary Methods

Deconvolution model for quantifying EV expression

To estimate the abundance of EV, we developed a deconvolution model to compute EV score using expression of five canonical markers, *CD63, CD9, CD81, TSG101and Alix*. Specifically, we constructed a rectangular matrix of normalised gene expression data, where each gene as a row and a sample as a column. For a *m* * *n* matrix denoted as $A_{m\times n}$, *m* is the total number of gene markers and *n* is the total number of samples. Since *m* is not equal to *n*, our model expanded Singular Value Decomposition (SVD) algorithm (**Reference1**:https://www.jstor.org/stable/2949777?seq=3#metadata_info_tab_contents) to solve the matrix as below

$$A_{m\times n} = U_{m\times m}\Sigma_{m\times n}V^T{}_{n\times n}$$

Where $V^T{}_{n\times n}$ is the transposed matrix of $V_{n\times n}$. $U_{m\times m}$ represents the eigenvectors between *A* and its transposed matrix, A^T. $V_{n\times n}$ is the eigenvectors between matrices A^T and *A***.** The singular values, the diagonal entries of the $\Sigma_{m\times n}$ matrix, are square roots of eigenvalues from AA^T or A^TA in a descending order. In the model, we only kept the maximum diagonal entries for the first column of $\Sigma_{m\times n}$ and set the remaining values to 0. Thus only the representative genes with high eigenvalues will be taken into account with accurate weights, avoiding the false rate caused by artificial selection. We further calculated the average value for each column, which was an estimator of the EV abundance in each sample. The Kruskal test, a single-factor comparative method, was subsequently used to examine the differences of EV abundance between multiple molecular subtypes and NT samples.

Composition of 22 immune cell fractions estimated using CIBERSORT

To gain further insights into the effect of EV abundance on immune cell population, we assessed immune cell fractions in all samples using CIBERSORT (**Reference2:** https://doi.org/10.1038/nmeth.3337). We performed 1000 iterations to compute relative proportions of 22 types of immune cells using the normalised gene expression data. Other parameters were selected as default. For each sample, the confidence of deconvolution accuracy was estimated by a global P value in the CIBERSORT. Only those samples with P values less than 0.05 were used in the subsequent analysis.

In order to compare cell fractions in relation to the abundance of EV, we computed quantiles of EV scores across the samples. We classified samples into high and low EV abundant groups based on their EV scores greater than 75% or less than 25% quantile.
The wilcox test was used to compare the difference between immune cell types in high and low EV abundant groups. R 4.0.3 (https://www.R-project.org/) was used in all statistical analyses.

Reference

1. Golub, G., and W. Kahan. "Calculating the Singular Values and Pseudo-Inverse of a Matrix." Journal of the Society for Industrial and Applied Mathematics: Series B, Numerical Analysis 2, no. 2 (1965): 205-24. Accessed March 31, 2021. http://www.jstor.org/stable/2949777.

2.Newman, A., Liu, C., Green, M. et al. Robust enumeration of cell subsets from tissue expression profiles. Nat Methods 12, 453–457 (2015). https://doi.org/10.1038/nmeth.3337
